# Association of AFP with Hepatocellular Carcinoma Subtypes

**DOI:** 10.3390/cancers18213524

**Published:** 2026-11-02

**Authors:** Callie Rose, James Hosner, Seung Hwan Chung, Ling Wang, Linda Qu

**Affiliations:** 1 College of Human Medicine, Michigan State University, East Lansing, MI 48824, USA; hosnerja@msu.edu (J.H.); chungs17@msu.edu (S.H.C.); wangli35@msu.edu (L.W.); qutong@msu.edu (L.Q.); 2 Department of Medicine, Michigan State University, East Lansing, MI 48824, USA; 3 Department of Surgery, Michigan State University, East Lansing, MI 48823, USA

**Keywords:** alpha-fetoprotein, hepatocellular carcinoma, fibrolamellar, scirrhous, spindle-cell, clear-cell, pleomorphic

## Abstract

**Background/Objectives:** Alpha-fetoprotein (AFP) is often elevated in hepatocellular carcinoma (HCC) and has been linked to poor prognosis. Among HCC subtypes, the association is less defined. We aimed to characterize AFP and their association with prognosis for the fibrolamellar, scirrhous, clear-cell, spindle-cell, and pleomorphic subtypes of HCC. **Methods:** Patients in the National Cancer Database (NCDB) diagnosed with HCC between 1998–2021, including not otherwise specified (NOS), fibrolamellar, scirrhous, clear-cell, spindle-cell, and pleomorphic were included. Elevated AFP was defined as 400 ng/mL or above. Associations between clinical characteristics, including AFP levels, and HCC subtypes were analyzed using two-sample *t*-test, chi-square, and Fisher exact tests. Survival was assessed using Kaplan–Meier curves with log-rank tests. **Results:** The study population included 168,470 patients, of which 166,778 (99.0%) were HCC NOS, followed by 872 (0.5%) clear-cell, 417 (0.2%) fibrolamellar, 251 (0.2%) scirrhous, 136 (0.1%) spindle-cell, and 16 (0.01%) pleomorphic. Elevated AFP was highest in the spindle-cell subtype (30.9%), followed by HCC NOS (29.4%), scirrhous (25.1%), clear-cell (23.4%), and fibrolamellar (15.6%) (*p* < 0.0001). Elevated AFP was significantly different across stage of disease with elevation in 11.9% of fibrolamellar, 6.8% of scirrhous, 35.7% of spindle-cell, 25.2% of clear-cell, and 17.6% of HCC NOS at stage 2; 22.5%, 50%, 33.3%, 28.2%, and 44.2%, respectively at stage 3; and 13.8%, 30.4%, 31.7%, 42.2%, and 53.2%, respectively, at stage 4 (all *p* < 0.005). Elevated AFP was associated with decreased survival for the fibrolamellar (9.7 months when elevated vs. 39 months when not elevated), scirrhous (3.8 vs. 24.2) and clear-cell (7.2 vs. 25.9) subtypes (*p* < 0.0001). **Conclusions:** Elevated AFP is least prevalent for fibrolamellar HCC, including when stratifying by disease stage, though it is associated with worse survival in that population. AFP elevation may offer important prognostic information when rare subtypes are diagnosed.

## Simple Summary

Hepatocellular carcinoma (HCC) has several rare subtypes that each have characteristic diagnostic features, presentations, and prognosis. Alpha-fetoprotein (AFP) is often elevated in HCC and has been associated with poorer prognosis when elevated at diagnosis of HCC. This association has not been characterized for the rare subtypes of HCC. This retrospective study aimed to investigate AFP levels and its association with prognosis in the fibrolamellar, scirrhous, clear-cell, spindle-cell, and pleomorphic subtypes of HCC. The fibrolamellar, scirrhous, and clear-cell subtypes showed poorer prognosis when AFP was elevated at diagnosis. These findings suggest that AFP may provide prognostic information for these subtypes and that the subtype of HCC should be considered when interpreting AFP.

## 1. Introduction

Hepatocellular carcinoma (HCC) is a rare cancer, accounting for 2% of new cancer cases in the US [1]. Despite advancements in screening, diagnosis, and treatment, HCC has a significant mortality rate, with only 21.9% of patients expected to survive five or more years following diagnosis [1]. The 2019 WHO classification recognizes eight distinct subtypes of HCC: fibrolamellar, scirrhous, macrotrabecular massive, clear-cell, steatohepatitic, chromophobe, neutrophil-rich, and lymphocyte-rich, each of which has characteristic histological features, presentation, and prognosis [2]. Fibrolamellar is a rare but well-studied subtype that is believed to have distinct pathogenesis from HCC and other subtypes. Fibrolamellar presents in younger patients, frequently in the second and third decades of life, with no gender bias. Patients often have no history of cirrhosis or chronic liver disease [3]. Scirrhous is morphologically similar to fibrolamellar but presents in older patients with a history of cirrhosis [4]. Scirrhous tumors are clinically and pathologically aggressive with infiltrative growth patterns and frequent vascular invasion [4]. Clear-cell is a highly differentiated variant of HCC with similar etiology. It can present in patients with either cirrhotic or non-cirrhotic livers [5]. In comparison to HCC NOS, clear-cell typically has a more favorable prognosis [6]. Spindle-cell is a sarcomatous variant of HCC [7]. Spindle-cell often presents as larger and more poorly differentiated tumors with higher levels of extrahepatic and nodal metastasis [8]. Pleomorphic is a very rare sarcomatoid subtype of HCC that presents with large, poorly or undifferentiated tumors with associated poorer prognosis [9].

Alpha-fetoprotein (AFP) is a plasma protein produced by the liver that is often elevated in HCC. An AFP level of greater than 400 ng/mL is highly suggestive of HCC [10]. However, diagnosis requires specific imaging or histological criteria and cannot be made utilizing AFP alone. AFP elevation has also been correlated with higher rates of recurrence and worse prognosis in several studies [11,12,13]. The relationship between AFP and prognosis in the individual histological subtypes of HCC, however, has not been fully characterized.

The objectives of this study were to determine whether trends in AFP differ across the fibrolamellar, scirrhous, clear-cell, spindle-cell, and pleomorphic subtypes of HCC, as well as to characterize the relationship between AFP and prognosis in each subtype.

## 2. Materials and Methods

### 2.1. Study Population

**Figure 1:**
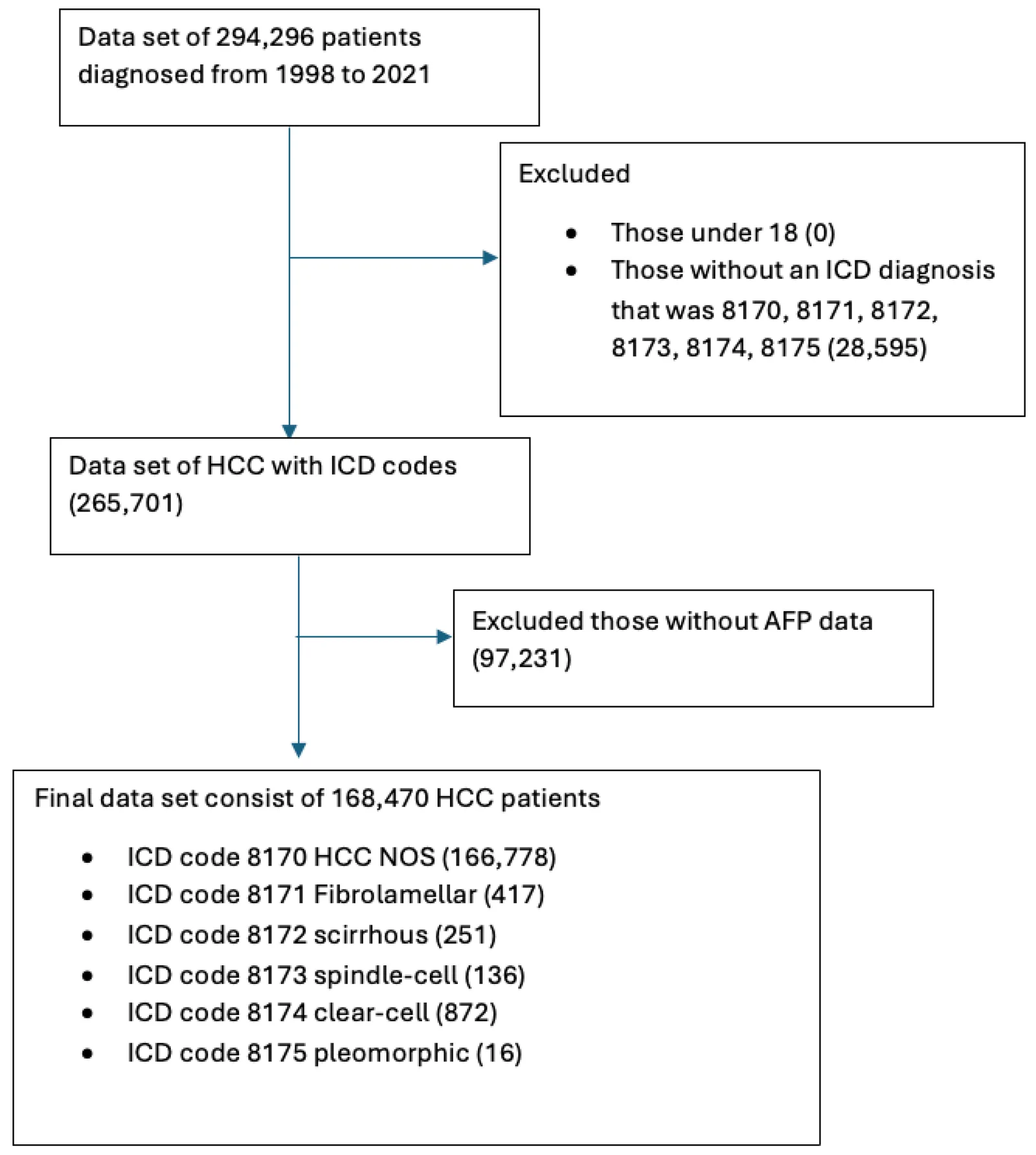
Stepwise extraction process from acquirement of the database to the creation of the final dataset.

Patients with hepatocellular carcinoma and recorded AFP values were identified from the National Cancer Database (NCDB). The NCDB captures patient information from approximately 1500 accredited hospitals across the United States and includes patients diagnosed with cancer between 1998 and 2021 [14]. Prior to the 2019 WHO reclassification, fibrolamellar, scirrhous, clear-cell, spindle-cell, and pleomorphic were recognized as HCC subtypes. As such, these are the subtypes predominantly described in the NCDB and thus included in this study. Hepatocellular carcinoma subtypes were identified according to the International Classification of Diseases for Oncology (ICD-O-3) codes [15]. ICD codes 8170 (HCC, NOS), 8171 (fibrolamellar), 8172 (scirrhous), 8173 (spindle-cell), 8174 (clear-cell), and 8175 (pleomorphic) were included in the study. Patients were excluded if they were 17 years old or younger, did not have a diagnosis of HCC as classified above, or did not have an AFP level recorded. Figure 1 shows the step-by-step process of obtaining the final dataset.

### 2.2. AFP Categorization

In this study, elevated AFP was defined as greater than or equal to 400 ng/mL. Those with AFP levels < 400 ng/mL were classified as having non-elevated AFP levels. AFP levels were reported separately to show the distinction between categorical and continuous data. For demographics and morbidity analysis, the data was combined to create groups of elevated AFP (greater than or equal to 400 ng/mL) and non-elevated AFP (<400 ng/mL).

In the NCDB, AFP levels were reported categorically through the year 2017 and continuously thereafter. For cases diagnosed in 1998 through 2017, AFP was obtained from the Collaborative Stage Site-Specific Factors variable CS_SITESPECIFIC_FACTOR_3, which recorded the highest pretreatment AFP value within predefined ranges which can be found in the Appendix A in Table A1 [16]. Subjects with AFP values in categories including values up to 399 ng/mL were included in the <400 ng/mL group and those with AFP, values 400 ng/mL or greater were included in the ≥400 ng/mL group. For patients diagnosed in or after 2018, Collaborative Stage Site-Specific Factors were no longer utilized, and the corresponding data was collected via the Site-Specific Data items, which provided a pretreatment AFP as a continuous rather than categorical variable [17]. AFP groups for this data were created by assigning all pretreatment AFP values of 399 ng/mL or less into the <400 ng/mL group and all values of 400 ng/mL or greater into the ≥400 ng/mL group. AFP was harmonized across eras by defining a common binary variable of <400 ng/mL versus ≥400 ng/mL. The proportion of patients classified as having AFP ≥400 ng/mL was 30.6% in the 1998–2017 era and 27.3% in the 2018–2021 era. While there is a significant difference in elevated AFP between eras (*p* < 0.001), the absolute difference is minimal. All final conclusions from the lifestyle analysis showed no change when each era was utilized independently.

### 2.3. Statistical Analysis

Per the data agreement policy with the NCDB, data cells with fewer than 10 cases were labeled as <10. Cell suppression was done by the database to protect the patient’s confidentiality in the form of providing completely deidentified data. Continuous variables are reported as mean with standard deviation. Discrete variables are reported as counts with percentages. For continuous variables, statistical analysis was performed utilizing a two-sample Student’s *t*-test. For categorical variables, a chi-square test or Fisher exact test was used, with applicable conditions applied based on what was appropriate. Survival analysis was conducted via the Kaplan–Meier test. Kaplan–Meier survival curves were generated and compared using the log-rank test. The endpoint of the Kaplan–Meier survival curve was overall survival, as an analysis utilizing cancer-specific survival was not possible due to limitations of the dataset. Overall survival was measured in months from diagnosis to death or last contact utilizing the variable DX_LASTCONTACT_DEATH_MONTHS from the dataset. Death from any cause was considered the event, while patients alive at last contact were censored at their last known follow-up. Survival analyses were limited to patients diagnosed between 2004 and 2020. Survival and follow-up information were not available for those diagnosed in 2021 and were thus excluded in the survival analysis, though were included in descriptive analyses. Multivariable Cox proportional hazard regression analyses were performed for each subtype separately. Models included AFP status, age, sex, treatment modality, tumor size, and stage of cancer as covariates. Hazard ratios (HRs) with 95% confidence intervals (CIs) were calculated. Stage 1 cancer, no treatment, size of <20 mm, female sex, and AFP <400 ng/mL served as reference groups for this analysis. Statistical significance was defined as *p* < 0.05. Results were considered statistically significant when *p* < 0.05. The analysis was performed using SAS software, Version 9.4 (TS1M9) of the SAS System. © 2025 SAS Institute Inc. [18].

## 3. Results

### 3.1. Subtype Prevalence and Demographics in Study Population

The study population consisted of 168,470 patients, of whom 166,778 (99.0%) were HCC NOS, 417 (0.2%) fibrolamellar, 251 (0.2%) scirrhous, 136 (0.1%) spindle-cell, 872 (0.5%) clear-cell, and 16 (0.01%) pleomorphic (Table 1). Of the subtypes, fibrolamellar and clear-cell were the most common. Patients with fibrolamellar were significantly younger (mean age 41.8 ± 21.4 years compared to 63.5 ± 11.0 for scirrhous, 65.7 ± 11.9 for spindle-cell, 67.1 ± 11.6 for clear-cell, 67.6 ± 10.2 for pleomorphic, and 64.8 ± 10.2 for HCC NOS (*p* < 0.0001)). Each subtype predominantly presented in men (61.9% of fibrolamellar, 72.5% of scirrhous, 76.5% of spindle-cell, 66.6% of clear-cell, 81.3% of pleomorphic, and 76.3% of HCC NOS (*p* < 0.0001)). The majority of patients with each subtype were white (79.1% fibrolamellar, 75.7% scirrhous, 77.2% spindle-cell, 76.7% clear-cell, 68.8% pleomorphic, and 73.8% HCC NOS). Primary payor was different between subtypes, with fibrolamellar having significantly more patients utilizing private insurance (51.1%) compared to 33.5% in scirrhous, 29.4% in spindle-cell, 26.1% in clear-cell, and 27.2% of HCC NOS (*p* < 0.05). Medicare was utilized as primary payor for more patients with scirrhous (47.7%), spindle-cell (46.3%), clear-cell (57.1%), pleomorphic (68.8%), and HCC NOS (49.6%) compared to significantly fewer patients with fibrolamellar (23.7%) (*p* < 0.05).

### 3.2. Comorbidities

The Charlson–Deyo Score, also referred to as the Charlson Comorbidity Index, assesses a patient’s comorbidities in order to predict mortality risk. A score of zero indicates no comorbidities whereas a score of 3 or more indicates a significant comorbidity burden. As shown in Table 1, fibrolamellar had significantly more (*p* < 0.05) patients with a score of zero (71.7%) compared to scirrhous (48.6), spindle-cell (55.1%), clear-cell (46.0), pleomorphic (43.8%), and HCC NOS (44.3%). Conversely, fibrolamellar had the fewest (8.9%) patients with a score of 3 or more compared to 17.9% scirrhous, 18.4% spindle-cell, 14.8% clear-cell, 18.8% pleomorphic, and 21.4% HCC NOS.

### 3.3. Included vs. Excluded Subjects

Patients were included in the analysis if a recorded AFP value at diagnosis was present and were excluded if the AFP value was absent. There were 168.470 patients with a recorded AFP value that were included and 97,231 patients without a recorded AFP value that were excluded. Table 2 compares demographic, clinical, and survival characteristics of the included versus excluded patients in the dataset. Age was nearly identical between included and excluded patients despite being significantly different (*p* < 0.0001). There was no significant difference in proportions of sex between the included and excluded groups. There were significant differences (*p* < 0.001) in percentages of patients with each subtype between excluded and included subjects; however, the differences were small (Cramer’s V = 0.0195). Excluded patients had significantly higher percentages of patients with unknown stage of disease (22.19%) in comparison to included patients (8.66%) (*p* < 0.0001). Race was very similar between the included and excluded groups, although differences were significant (*p* < 0.0001). Charlson–Deyo score was significantly different between groups with the excluded patients having a higher percentage with a score of 0 (53.3%) and a lower percentage with a score of 3 or more (14.99%) than the included patients (44.4%, 21.37%) (*p* < 0.001). Excluded patients had a significantly higher percentage without a recorded treatment status (58.11%) compared to included patients (0.92%) (*p* < 0.0001). Percentages of patients with no primary-site surgery were similar but significantly different between groups with 70.92% of excluded patients and 73.83% of included patients not having surgical treatment (*p* < 0.0001). Significant differences in the modality of chemotherapy were present between groups with more included patients having single-agent chemotherapy (29.16%) than excluded patients (17.88%) (*p* < 0.0001). Categories for survival analysis were significantly different with the percentage of evaluable N being 97.35% in excluded patients and 91.41% in included patients (*p* < 0.0001). Median observed survival months differed significantly between groups, with survival being shorter in excluded patients (11.37, 95% CI 11.17–11.56) than in included patients (16.53, 95% CI 16.33–16.76).

### 3.4. Stage

Significant differences in cancer stage at presentation were noted between fibrolamellar, spindle-cell, and HCC NOS (*p* < 0.05). At stage 1 and stage 2, more patients had HCC NOS (33.3% and 20.6%) in comparison to fibrolamellar (26.1 and 10.2%) and spindle-cell (19.1% and 10.3%). Patients presented with stage 3 disease at similar rates across subtypes. At stage 4, fewer patients had HCC NOS (16.8%) compared to fibrolamellar (38.1%) and spindle-cell (44.1%). Due to its small sample size, the pleomorphic subtype was not included in this analysis.

### 3.5. AFP

When data was collected categoricaly in 1998 to 2017, median AFP for patients was the 1–19 ng/mL category across subtype groups. In 2018 and beyond when AFP was collected continuously, mean AFP (ng/mL) was 1555 ± 3253 for HCC NOS, 1042 ± 2777 for fibrolamellar, 1452 ± 3122 for scirrhous, 1332 ± 3141 for spindle-cell, and 812 ± 2407 for clear-cell (*p* = 0.001).

Table 3 outlines the percent of patients with each subtype that had elevated AFP levels (defined as greater than or equal to 400 ng/mL). Elevated AFP was seen in 15.6% of fibrolamellar, 25.1% of scirrhous, 30.9% of spindle-cell, 23.4% of clear-cell, 18.8% of pleomorphic, and 29.4% of HCC NOS (*p* < 0.0001).

### 3.6. AFP and Stage of Disease

There was no significant difference across subtypes for stage 1 disease. At stage 2, more patients with spindle-cell and clear-cell had elevated AFP (35.7%, 25.2%) in comparison to fibrolamellar (11.9%), scirrhous (6.8%), and HCC NOS (17.6%) (*p* = 0.0054). At stage 3, more patients with scirrhous and HCC NOS had elevated AFP (50%, 44.2%) compared to fibrolamellar (22.5%), spindle-cell (28.2%), and clear-cell (28.2%) (*p* < 0.0001). At stage 4, HCC NOS and clear-cell had the highest numbers (53.2%, 42.2%) of patients with elevated AFP compared to fibrolamellar (13.8%), scirrhous (30.4%), and spindle-cell (31.7%) (*p* < 0.0001). Pleomorphic was not included in this analysis due to a small sample size.

**Figure 2:**
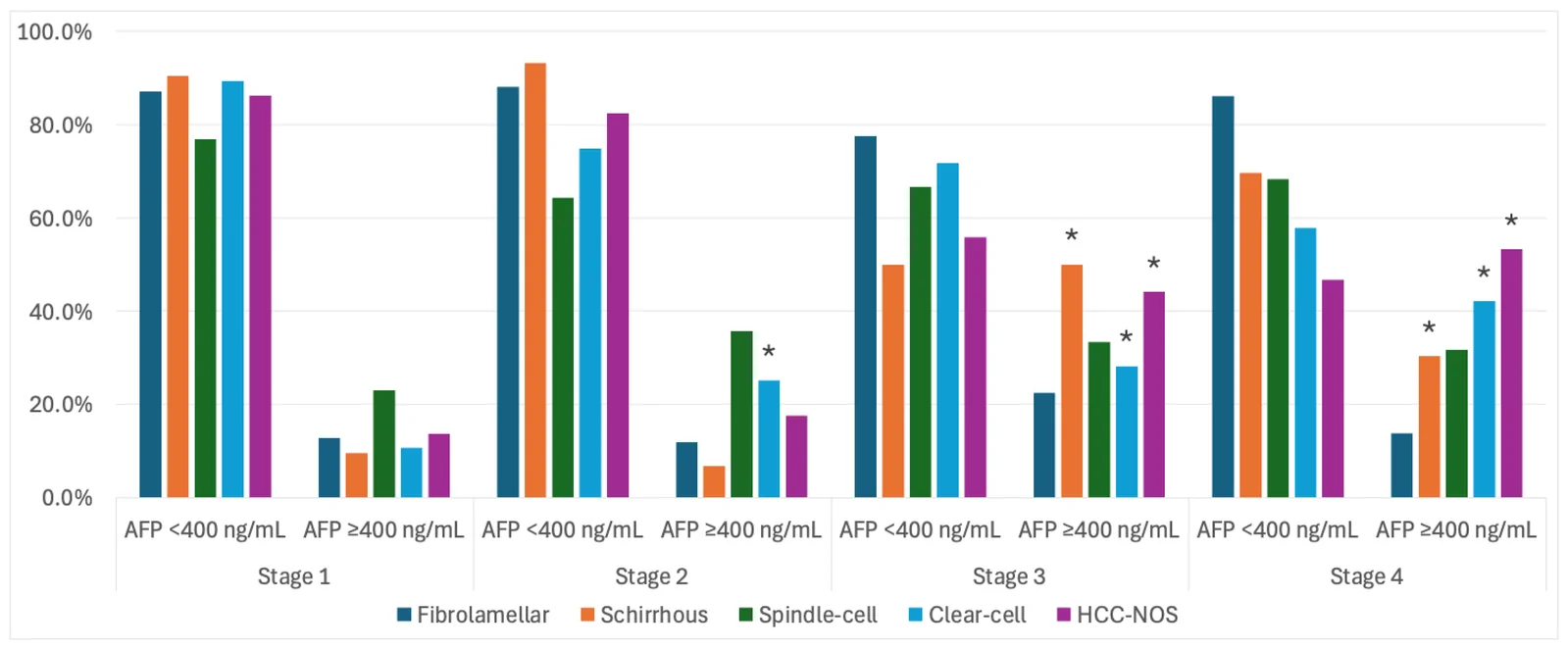
Comparison of AFP levels in HCC subtypes when stratified by stage of disease. * *p* < 0.05 when proportions of elevated and non-elevated AFP are compared to HCC NOS.

Figure 2 compares the percentages of patients with each subtype that have non-elevated and elevated AFP at each stage of disease. Clear-cell and HCC NOS both show increasing percentages of patients with elevated AFP as the stage of disease increases. Clear-cell has 10.7% at stage 1, 25.2% at stage 2, 28.2% at stage 3, and 42.2% at stage 4 and HCC NOS has 13.7%, 17.6%, 44.2%, and 53.2%, respectively. Fibrolamellar, scirrhous, and spindle-cell show no trend in the percentage of patients with elevated AFP associated with stage of disease.

### 3.7. AFP and Survival

**Figure 3:**
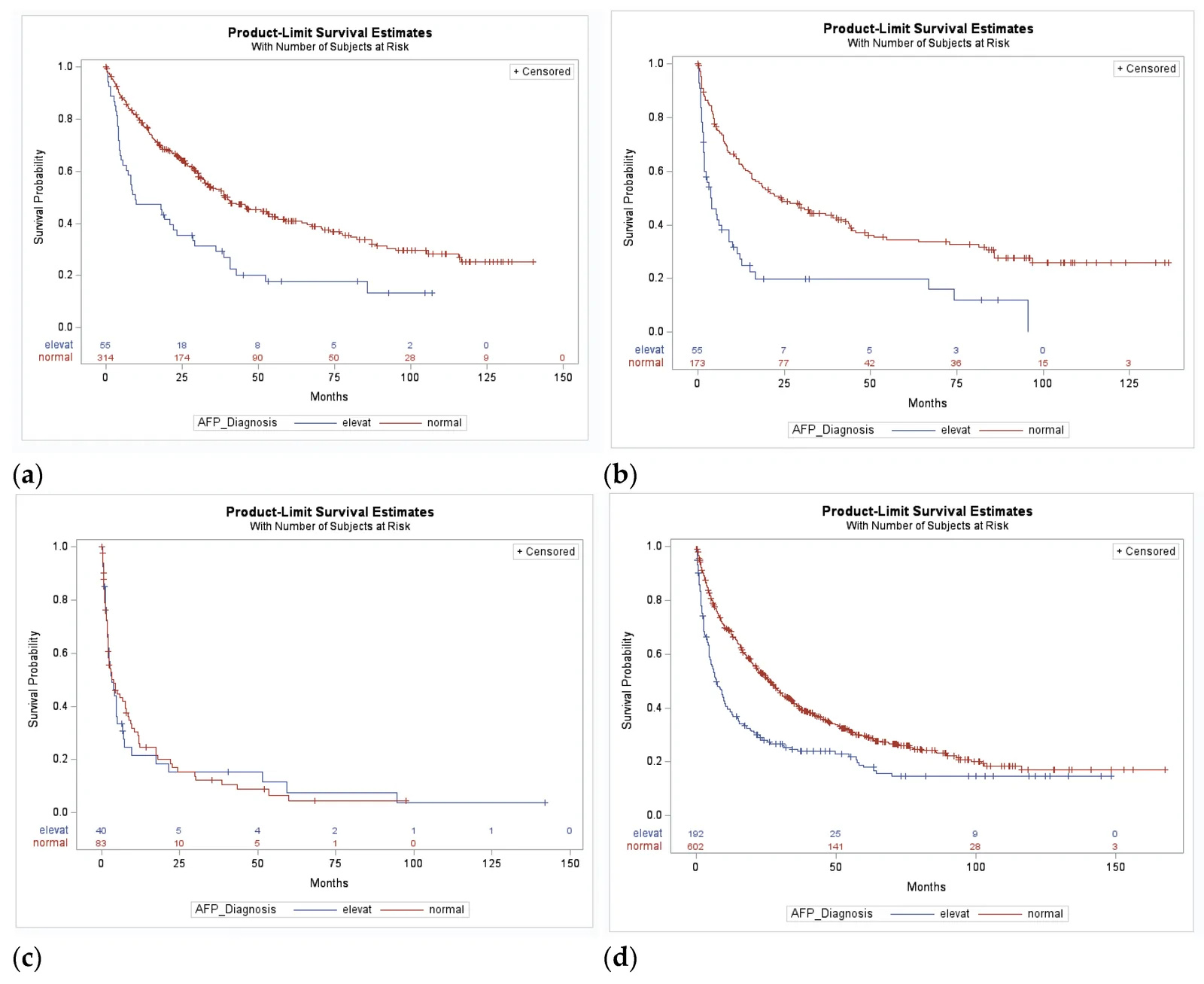
Kaplan–Meier survival curves with number at risk for each HCC subtype when AFP is elevated vs. non-elevated (**a**) Fibrolamellar HCC (*p* < 0.0001); (**b**) scirrhous HCC (*p* < 0.0001); (**c**) spindle-cell HCC (*p* = 0.84); (**d**) clear-cell HCC (*p* < 0.0001).

When AFP was elevated, there were significant differences in median survival for the fibrolamellar, scirrhous, and clear-cell subtypes (Figure 3). Survival for fibrolamellar patients with elevated AFP was 9.7 months (95% CI 4.90–23.39) vs. 39 months (CI 32.33–52.83) when AFP was non-elevated (*p* < 0.0001). For scirrhous, median survival was 3.8 months (CI 1.81–8.94) vs. 24.2 months (CI 15.61–40.15) (*p* < 0.0001). For clear-cell, median survival was 7.2 months (CI 5.19–9.89) when AFP was elevated and 25.9 months (CI 21.82–29.77) when AFP was non-elevated (*p* < 0.0001). Spindle-cell interestingly showed a median survival of 3.7 months (CI 2.04–7.49) when AFP was elevated in comparison to 3.2 months (CI 1.74–5.09) when AFP was non-elevated, although this was not significant (*p* = 0.84). Table 4 outlines median follow-up for each subtype of HCC. Median follow-up was estimated using the reverse Kaplan–Meier method. Among the full dataset, the median follow-up was 47.4 months (95% CI 46.9–47.7 months). To address potential confounding by tumor stage, a stage-specific Kaplan–Meier analysis was performed as outlined in Table 5. Significant survival differences persisted for some subtypes at certain stages. Fibrolamellar showed significantly lower survival when AFP was elevated at stages III (*p* = 0.0076) and IV (*p* < 0.0001), scirrhous at stage I (*p* < 0.0001), and clear-cell at stages I (*p* = 0.026) and IV (*p* = 0.0029). Survival continued to show no significant differences for spindle-cell when stratified by stage of disease. Treatment-stratified analysis was also performed between surgical and non-surgical management and is outlined in Table 6. Further stratification by stage was not possible due to the rarity of the subtypes, producing a small sample size for certain groups. Significantly lower survival was present when AFP was elevated for non-surgical management in fibrolamellar (*p* = 0.0014), scirrhous (*p* = 0.0082), and clear-cell (*p* < 0.0001). There was no significant difference in survival for non-surgical management in spindle-cell. No significant differences in survival were present for surgical management across subtypes.

Table 7 outlines an additional analysis utilizing multivariable Cox proportional hazards regression that was performed for the fibrolamellar, spindle-cell, clear-cell, and scirrhous subtypes of HCC to adjust for variables that may influence survival. After adjustment for these covariates, elevated AFP remained significantly associated with increased mortality in patients with fibrolamellar (adjusted hazard ratio [aHR] = 1.87, 95% confidence interval [CI]: 1.26–2.76; *p* = 0.0019) and clear-cell (aHR = 1.35, 95% CI: 1.10–1.66; *p* = 0.0036). Elevated AFP was not significantly associated with increased mortality in spindle-cell (aHR = 1.17, 95% CI: 0.71–1.93; *p* = 0.53) or scirrhous (aHR = 1.22, 95% CI: 0.78–1.90; *p* = 0.38). Although the point estimates indicated an increased mortality hazard, the confidence intervals included the null value, suggesting that the available evidence was insufficient to establish statistically significant associations in these subtypes.

## 4. Discussion

Hepatocellular carcinoma (HCC) is not a single biologic entity, and the rare histologic subtypes included in this study appear to differ in both frequency and prognostic significance of alpha-fetoprotein (AFP) elevation. In this national cohort, AFP ≥ 400 ng/mL was most common in spindle-cell HCC (30.9%), followed by HCC NOS (29.4%), scirrhous HCC (25.1%), clear-cell HCC (23.4%), pleomorphic HCC (18.8%), and fibrolamellar HCC (15.6%). However, the estimate for pleomorphic HCC should be interpreted cautiously given the small number of cases. The prognostic value of AFP also appeared to vary by subtype. Elevated AFP was associated with worse survival in fibrolamellar, scirrhous and clear-cell HCC, but AFP elevation was not associated with a significant difference in survival in spindle-cell HCC. These findings suggest that AFP should not be interpreted as a uniform marker across all HCC histology. Rather, AFP appears most useful when interpreted alongside histology and disease stage.

AFP is better understood as a marker of tumor phenotype and hepatocellular secretory activity than as a stand-alone diagnostic test. Current AASLD guidance emphasizes that HCC diagnosis should not rely on AFP alone and that imaging and/or pathology remain central to diagnosis depending on the clinical context [19]. This distinction is important for the present study. Although AFP ≥ 400 ng/mL has historically been used as a high-specificity threshold, it is clinically more appropriate to interpret this cutoff as a high-risk biomarker threshold rather than a diagnostic definition of HCC. Prior studies showed that AFP-positive HCC is associated with aggressive tumor biology, including vascular invasion, greater tumor burden, and worse outcomes, but our findings suggest that these relationships may not apply equally to every histologic variant [20].

The fibrolamellar findings are particularly important because they illustrate the limitations of using AFP as a general HCC marker. Fibrolamellar HCC had the lowest rate of AFP elevation in this cohort, which fits with its distinct clinical and molecular profile. Unlike HCC NOS, fibrolamellar carcinoma often arises in younger patients without cirrhosis or chronic liver disease and is closely linked to the DNAJB1-PRKACA fusion oncogene [21,22]. The low frequency of AFP elevation in fibrolamellar HCC is consistent with prior reports that AFP is typically not elevated in this subtype, although the biologic basis for this pattern remains incompletely understood [23,24]. However, when AFP elevation is present in fibrolamellar HCC, it may identify a higher-risk subgroup. In our analysis, fibrolamellar patients with AFP ≥ 400 ng/mL had significantly worse survival. After adjustment for covariates, AFP ≥ 400 ng/mL remained significantly associated with poorer prognosis, suggesting that elevated AFP may provide prognostic information beyond the demographic and clinical characteristics included in the covariate analysis. These findings are consistent with a previous study that found elevated AFP was associated with worse overall survival among patients with fibrolamellar carcinoma undergoing curative resection [25]. When stratified by stage of disease, significantly worse survival when AFP was elevated remained for stages III and IV. In this study, a majority of patients with fibrolamellar HCC presented with stage II or IV disease. This is consistent with a previous study that described fibrolamellar HCC presenting at a later stage of disease [24]. While AFP is not commonly elevated in fibrolamellar HCC, it may indicate a later stage of disease and carry a heavier prognostic weight when elevated. This distinction should be emphasized because while an AFP level < 400 ng/mL cannot exclude aggressive fibrolamellar disease, an elevated AFP may suggest a more biologically unfavorable phenotype.

Clear-cell HCC showed a different pattern. Although clear-cell tumors had a lower overall frequency of AFP elevation than HCC NOS, AFP elevation increased with advancing stage and was associated with worse survival, which remained true following survival adjustments and for stage I and IV when stratified by stage of disease. Prior data regarding AFP in clear-cell HCC are limited. Yang et al. reported a mean AFP of 1276.4 ng/mL in a series of 13 patients [6]. Among the rare subtypes in this study, clear-cell HCC therefore appeared to behave most like HCC NOS with respect to AFP. This is biologically plausible because clear-cell HCC is often regarded as a morphologic variant of HCC NOS rather than a biologically distinct disease process. Population-based work has shown that clear-cell HCC overlaps substantially with HCC NOS in clinical behavior after accounting for stage, grade, and treatment variables [9,26]. A separate SEER-based nomogram study of primary clear-cell carcinoma of the liver also supports that clear-cell HCC shares important prognostic determinants with HCC NOS [27]. Taken together with our results, these findings suggest that AFP-positive clear-cell HCC may represent a more aggressive, HCC NOS-like subgroup in which AFP elevation is associated with advancing disease and poorer prognosis.

Scirrhous HCC also demonstrated clinically meaningful AFP elevation, with 25.1% of patients having AFP ≥ 400 ng/mL. Prior literature has suggested that AFP elevation may occur relatively frequently in this subtype. Murtha-Lemekhova et al. reported AFP greater than 20 IU/mL in 70.3% of patients with scirrhous HCC [28]. Direct comparison with the present study is limited, however, because that study used a substantially lower AFP threshold than the AFP ≥ 400 ng/mL cutoff used in our analysis. In the present cohort, elevated AFP was associated with markedly worse survival, with median survival of 3.8 months compared with 24.2 months among patients with AFP < 400 ng/mL. When stratified by stage of disease, this significant difference persisted for patients with stage I disease. These findings suggest that marked AFP elevation may identify a subgroup of scirrhous tumors with unfavorable prognosis, particularly in patients presenting with early-stage disease. These survival differences did not persist following covariate analysis, with hazard ratios being insignificant. Given the rarity and heterogeneous clinical features of scirrhous HCC, additional studies are needed to determine whether AFP provides prognostic information independent of stage, treatment, liver function, and other tumor characteristics.

Spindle-cell HCC demonstrated one of the highest frequencies of AFP elevation in the study, with AFP ≥ 400 ng/mL in 30.9% of patients. Prior evidence regarding AFP in spindle-cell HCC has largely been restricted to small case series. Maeda et al. reported AFP greater than 20 IU/mL in six of 13 patients and greater than 100 IU/mL in three patients [7]. Although spindle-cell tumors had one of the highest rates of AFP elevation in this cohort, AFP did not significantly influence survival, and this remained true when stratified by stage of disease and following covariate analysis. This may reflect the aggressive biology of spindle cells, in which prognosis is poor regardless of AFP status. Prior studies have characterized the subgroup at an advanced stage, with atypical imaging features, a high recurrence risk, and poorer outcomes compared with HCC NOS [29,30]. Clinically, this supports that AFP-negative disease should not be considered reassuring in such rare HCC variants with aggressive histology.

The interpretation of AFP in pleomorphic HCC remains particularly limited. Only 16 patients with pleomorphic HCC were identified in this cohort, of whom three (18.8%) had AFP ≥ 400 ng/mL. Previous evidence is similarly sparse. A SEER-based analysis of 17 pleomorphic HCC cases reported positive AFP in 76.5% of patients [9]. Differences in the definition of AFP positivity and the extremely small sample sizes limit direct comparison between these estimates. The present data therefore do not permit strong conclusions regarding the frequency or prognostic significance of AFP elevation in pleomorphic HCC.

Several limitations should be acknowledged in our study. First, this study is retrospective and based on NCDB data, which limits adjustment for important clinical variables that may have influenced treatment decisions and eventual prognosis. Second, AFP reports changed over time, with categorical reporting before 2017 and continuous reporting afterward, introducing measurement heterogeneity across the study period. Third, because patients without recorded AFP were excluded, selection bias is possible if AFP testing differed according to disease severity, treatment setting, institution or time period. Finally, rare subtypes such as pleomorphic and spindle-cell HCC had small sample sizes, limiting the ability to perform robust stage-specific or treatment-specific survival analyses.

Despite these limitations, this study provides one of the first national evaluations of AFP across rare HCC histologic subtypes and supports a subtype-specific approach to biomarker interpretation. The most clinically meaningful finding is not simply that AFP differs across subtypes, but that the prognostic capability of the tumor marker differs as well. AFP elevation appears to identify higher-risk patients with fibrolamellar and clear-cell HCC, while non-elevated AFP should not be interpreted as reassuring in aggressive subtypes such as spindle-cell HCC. The prognostic association observed in scirrhous HCC warrants further validation given the rarity of this subtype and the potential for unmeasured clinical and treatment-related confounding. Future studies incorporating additional biomarkers, liver function, treatment characteristics, and centralized pathology review may further improve risk stratification in rare HCC subtypes.

## 5. Conclusions

In this national cohort, AFP elevation varied significantly across histologic subtypes of HCC and was not uniformly associated with prognosis. AFP ≥ 400 ng/mL was least common in fibrolamellar HCC, but when present, it was associated with worse survival, suggesting that AFP elevation may identify a higher-risk subset within this otherwise AFP-low subtype. Clear-cell HCC demonstrated a more HCC NOS-like pattern, with AFP elevation increasing across advancing stages and being associated with worse survival. Additional analysis to adjust for demographic and clinical characteristics in the subtypes maintained a significantly lower survival in fibrolamellar and clear-cell HCC, strengthening the possible prognostic capability of elevated AFP in these subtypes. Elevated AFP was also associated with worse survival in scirrhous HCC. In contrast, spindle-cell HCC showed no significant AFP-based survival difference. Finally, interpretation of pleomorphic HCC remains limited by its very small sample size. Overall, these findings support a subtype-specific approach to AFP interpretation in which its prognostic value depends on the histological subtype rather than functioning as a uniform biomarker across all HCC subtypes.

## Figures and Tables

**Table 1:** Characteristics of the histological subtypes of HCC.

Variable	Fibrolamellar	Scirrhous	Spindle-Cell	Clear-Cell	Pleomorphic	HCC NOS
Prevalence	417 (0.2%)	251 (0.2%)	136 (0.1%)	872 (0.5%)	16 (0.01%)	166,778 (99.0%)
Age (mean, SD)	41.8 (21.4) *	63.5 (11.0)	65.7 (11.9)	67.1 (11.6) *	67.6 (10.2)	64.8 (10.2)
Sex	*			*		
Male	258 (61.9%)	182 (72.5%)	104 (76.5%)	581 (66.6%)	13 (81.3%)	127,198 (76.3%)
Female	159 (38.1%)	69 (27.5%)	32 (23.5%)	291 (33.4%)	<10	39,580 (23.7%)
Race				*		
White	330 (79.1%)	190 (75.7%)	105 (77.2%)	669 (76.7%)	11 (68.8%)	123,054 (73.8%)
Black	54 (12.9%)	39 (15.5%)	17 (12.5%)	85 (9.7%)	<10	25,617 (15.4%)
Other	29 (7.0%)	21 (8.4%)	13 (9.6%)	108 (12.4%)	<10	16,235 (9.7%)
Unknown	4 (1.0%)	<10	<10	10 (1.1%)	<10	1872 (1.1%)
Primary Payor	*			*		
Not Insured	19 (4.6%)	15 (6.0%)	6 (4.4%)	25 (2.9%)	<10	8409 (5.0%)
Private Insurance	213 (51.1%)	84 (33.5%)	40 (29.4%)	228 (26.1%)	<10	45,306 (27.2%)
Medicaid	75 (18.0%)	29 (11.6%)	22 (16.2%)	95 (10.9%)	<10	24,948 (15.0%)
Medicare	99 (23.7%)	119 (47.4%)	63 (46.3%)	498 (57.1%)	11 (68.8%)	82,777 (49.6%)
Other Government	<10	0	<10	17 (1.9%)	0	3117 (1.9%)
Status Unknown	<10	<10	<10	9 (1.0%)	0	2221 (1.3%)
Charlson–Deyo Score	*					
0	299 (71.7%)	122 (48.6%)	75 (55.1%)	401 (46.0%)	7 (43.8%)	73,899 (44.3%)
1	56 (13.4%)	63 (25.1%)	25 (18.4%)	238 (27.3%)	5 (31.3%)	38,340 (23.0%)
2	25 (6.0%)	21 (8.4%)	11 (8.1%)	104 (11.9%)	1 (6.3%)	18,777 (11.3%)
3 or more	37 (8.9%)	45 (17.9%)	25 (18.4%)	129 (14.8%)	3 (18.8%)	35,762 (21.4%)
NCDB Analytic Stage ^1^	*		*			
Stage 0	<10	<10	0 (0%)	0 (0%)	<10	<10
Stage 1	109 (26.1%)	84 (33.5%)	26 (19.1%)	309 (35.4%)	<10	55,494 (33.3%)
Stage 2	42 (10.1%)	44 (17.5%)	14 (10.3%)	163 (18.7%)	<10	34,296 (20.6%)
Stage 3	80 (19.2%)	48 (19.1%)	27 (19.9%)	188 (21.6%)	<10	34,467 (20.7%)
Stage 4	159 (38.1%)	56 (22.3%)	60 (44.1%)	147 (16.9%)	<10	28,001 (16.8%)
Unknown	27 (6.5%)	19 (7.6%)	<10	65 (7.5%)	<10	14,519 (8.7%)
Grade	*	*	*	*		
Grade 1	23 (5.5%)	20 (8.0%)	<10	77 (8.8%)	<10	11,221 (10.6%)
Grade 2	75 (18.0%)	50 (19.9%)	<10	211 (24.2%)	<10	16,556 (15.6%)
Grade 3	19 (4.6%)	21 (8.4%)	35 (25.7%)	70 (8.0%)	<10	7298 (6.9%)
Grade 4	<10	<10	<10	<10	<10	396 (0.4%)
Unknown	299 (71.7%)	157 (62.5%)	92 (67.6%)	510 (58.5%)	<10	70,710 (66.6%)
Lymphovascular Invasion	*	*	*	*		
No	110 (26.4%)	70 (27.9%)	24 (17.6%)	196 (22.5%)	<10	23,529 (14.1%)
Yes	68 (16.3%)	19 (7.6%)	10 (7.4%)	83 (9.5%)	<10	6273 (3.8%)
N/A	<10	<10	<10	<10	<10	435 (0.3%)
Unknown	237 (56.8%)	162 (64.5%)	102 (75.0%)	591 (67.8%)	12 (75.0%)	136,541 (81.9)%

* *p* < 0.05 when compared to HCC NOS. ^1^ NCDB Analytic Stage Group was defined by the NCDB as the reported pathologic stage group or clinical stage group if the pathological stage was not reported.

**Table 2:** Comparison of demographic and clinical chararacteristics in included vs. excluded Patients.

Characteristic	AFP Absent (Excluded)	AFP Present (Included)	*p*-Value
Age (mean, SD)	64.28 (11.77)	64.73 (10.27)	<0.0001
Age (median, IQR)	64 (56–73)	64 (58–71)	
Sex			0.088
Male	73,783 (75.88%)	128,336 (76.18%)	
Female	23,448 (24.12%)	40,134 (23.82%)	
Histology			<0.0001
HCC NOS	95,851 (98.58%)	166,778 (99.00%)	
Fibrolamellar	372 (0.38%)	417 (0.25%)	
Scirrhous	188 (0.19%)	251 (0.15%)	
Spindle-cell	105 (0.11%)	136 (0.08%)	
Clear-cell	690 (0.71%)	872 (0.52%)	
Pleomorphic	25 (0.03%)	16 (0.01%)	
NCDB Analytic Stage			<0.0001
Stage I	26,151 (26.90%)	56,027 (33.26%)	
Stage II	16,157 (16.62%)	34,561 (20.51%)	
Stage III	18,389 (18.91%)	34,813 (20.66%)	
Stage IV	14,923 (15.35%)	28,427 (16.87%)	
Unknown stage	21,573 (22.19%)	14,582 (8.66%)	
Other	38 (0.04%)	60 (0.04%)	
Race			<0.0001
White	72,182 (74.24%)	124,359 (73.82%)	
Black	14,103 (14.50%)	25,816 (15.32%)	
Other	9537 (9.81%)	16,407 (9.74%)	
Unknown	1409 (1.45%)	1888 (1.12%)	
Primary Payor			<0.0001
Not Insured	4765 (4.90%)	8475 (5.03%)	
Private Insurance	29,708 (30.55%)	45,874 (27.23%)	
Medicaid	12,236 (12.58%)	25,170 (14.94%)	
Medicare	45,467 (46.76%)	83,567 (49.60%)	
Other Government	2251 (2.32%)	3144 (1.87%)	
Status Unknown	2804 (2.88%)	2240 (1.33%)	
Charlson–Deyo Score			<0.0001
0	51,825 (53.30%)	74,803 (44.40%)	
1	22,186 (22.82%)	38,727 (22.99%)	
2	8643 (8.89%)	18,939 (11.24%)	
3 or more	14,577 (14.99%)	36,001 (21.37%)	
Treatment Status			<0.0001
Missing/blank	56,503 (58.11%)	1545 (0.92%)	
No treatment given	11,707 (12.04%)	32,474 (19.28%)	
Treatment given	27,486 (28.27%)	130,399 (77.40%)	
Active surveillance	388 (0.40%)	2073 (1.23%)	
Unknown	1147 (1.18%)	1979 (1.17%)	
Primary-Site Surgery			<0.0001
No primary-site surgery	68,959 (70.92%)	124,388 (73.83%)	
Chemotherapy			<0.0001
None	55,433 (57.01%)	88,884 (52.76%)	
Chemotherapy NOS	4260 (4.38%)	4687 (2.78%)	
Single-agent	17,386 (17.88%)	49,134 (29.16%)	
Multiagent	9265 (9.53%)	8908 (5.29%)	
Survival Analysis			<0.0001
Total Evaluable N	94,653	153,998	
Deaths	76,600 (80.9%)	109,381 (71.0%)	
Censored	18,053 (19.1%)	44,617 (29.0%)	
Median OS, months (95% CI)	11.37 (11.17–11.56)	16.53 (16.33–16.76)	

**Table 3:** AFP levels in the HCC subtypes.

	AFP Value	Fibrolamellar	Scirrhous	Spindle-Cell	Clear-Cell	Pleomorphic	HCC NOS
		*			*		
1998 to 2017	<400 ng/mL	209 (86%)	126 (75.9%)	55 (63.2%)	456 (74.6%)	<10	73,709 (69.4%)
	≥400 ng/mL	34 (14%)	40 (24.1%)	32 (36.8%)	155 (25.4%)	<10	32,472 (30.6%)
Total		243	166	87	611	15	106,181
		*			*		
2018 and beyond	<400 ng/mL	143 (82.2%)	62 (73%)	39 (79.6%)	212 (81.2%)	<10	44,063 (72.7%)
	≥400 ng/mL	31 (17.8%)	23 (27%)	10 (20.4%)	49 (18.8%)	<10	16,534 (27.3%)
Total		174	85	49	261	<10	60,597
		*			*		
1998 and beyond	<400 ng/mL	352 (84.4%)	188 (74.9%)	94 (69.1%)	668 (76.6%)	13 (81.2%)	117,772 (70.6%)
	≥400 ng/mL	65 (15.6%)	63 (25.1%)	42 (30.9%)	204 (23.4%)	<10	49,006 (29.4%)
Total		417	251	136	872	16	166,778

* *p* < 0.05 when compared to HCC NOS.

**Table 4:** Median follow-up for the subtypes of HCC.

	Survival N	Median Follow-Up Months (95% CI)
Fibrolamellar	369	62.3 (55.7–78.6)
Scirrhous	228	86.8 (78.8–95.1)
Spindle-cell	123	68.4 (40.5–141.9)
Clear-cell	794	70.2 (61.6–75.2)
Pleomorphic	Sample too small	Not estimable

**Table 5:** Median overall survival months when stratified by stage of disease and AFP.

	AFP Level	Stage I Median Survival Months	*p*-Value	Stage II Median Survival Months	*p*-Value	Stage III Median Survival Months	*p*-Value	Stage IV Median Survival Months	*p*-Value
Fibrolamellar	<400 ng/mL	76.7	0.3	80.2	0.76	32.3	0.0076	26.8	<0.0001
	≥400 ng/mL	38.7		28.3		8.6		4.2	
Scirrhous	<400 ng/mL	72.9	<0.0001	39	0.26	13.67	0.2045	6.14	0.4799
	≥400 ng/mL	11.5		12.19		5.65		4.07	
Spindle-Cell	<400 ng/mL	12	0.32	17.2	0.59	2.6	0.56	2	0.89
	≥400 ng/mL	40		9.4		3.2		1.97	
Clear-Cell	<400 ng/mL	47.6	0.026	34.6	0.29	10.6	0.25	8.51	0.0029
	≥400 ng/mL	30.7		22.6		7.5		2.279	

**Table 6:** Median overall survival for the HCC subtypes when stratified by surgical vs. non-surgical management.

	AFP Level	All Patients Median Survival Months	*p*-Value	Non-Surgical Management Median Survival Months	*p*-Value	Surgical Management Median Survival Months	*p*-Value
Fibrolamellar	<400 ng/mL	39	<0.0001	14.2	0.0014	87	0.21
	≥400 ng/mL	9.7		4.8		42	
Scirrhous	<400 ng/mL	24.2	<0.0001	8.1	0.0082	96.9	0.29
	≥400 ng/mL	3.8		2.7		66.7	
Spindle-cell	<400 ng/mL	3.2	0.84	2	0.4	17.3	0.6
	≥400 ng/mL	3.7		2		9.4	
Clear-cell	<400 ng/mL	25.9	<0.0001	12.5	<0.0001	63.1	0.97
	≥400 ng/mL	7.2		4.6		57.5	

**Table 7:** Adjusted survival and covariates for the HCC subtypes.

Covariates	Levels	Fibrolamellar HCC		Spindle-Cell HCC		Clear-Cell HCC		Scirrhous HCC	
		Hazard Ratio (95% CI)	*p*-Value	Hazard Ratio (95% CI)	*p*-Value	Hazard Ratio (95% CI)	*p*-Value	Hazard Ratio (95% CI)	*p*-Value
AFP Level	≥400 ng/mL vs. <400 ng/mL	1.87 (1.26–2.76)	0.0019	1.17 (0.71–1.93)	0.53	1.35 (1.10–1.66)	0.0036	1.22 (0.78–1.90)	0.38
Age		1.00 (0.995–1.012)	0.42	1.00 (0.978–1.017)	0.77	1.01 (1.005–1.021)	0.0009	1.01 (0.990–1.024)	0.44
Sex	Male	1.13 (0.85–1.51)	0.39	1.01 (0.60–1.71)	0.96	0.91 (0.76–1.09)	0.31	1.20 (0.82–1.76)	0.35
Stage of Disease	Stage II	1.19 (0.61–2.32)	0.6	0.91 (0.41–2.06)	0.83	1.22 (0.94–1.58)	0.13	1.53 (0.90–2.60)	0.12
	Stage III	2.04 (1.31–3.20)	0.0018	2.35 (1.05–5.24)	0.037	1.59 (1.24–2.05)	0.0003	1.99 (1.11–3.55)	0.02
	Stage IV	3.65 (2.32–5.74)	<0.001	2.26 (1.19–4.29)	0.013	2.55 (1.94–3.34)	<0.001	1.73 (1.00–3.01)	0.051
	Unknown	1.55 (0.82–2.94)	0.18	2.25 (0.83–6.09)	0.11	1.86 (1.30–2.64)	0.0006	4.04 (2.00–8.16)	<0.001
Treatment	Surgery only	0.096 (0.059–0.156)	<0.001	0.192 (0.103–0.357)	<0.001	0.161 (0.124–0.209)	<0.001	0.180 (0.104–0.313)	<0.001
	Radiation only	0.315 (0.183–0.540)	<0.001	0.201 (0.057–0.706)	0.012	0.377 (0.274–0.518)	<0.001	0.662 (0.361–1.215)	0.183
	Chemotherapy only	0.275 (0.174–0.434)	<0.001	0.307 (0.150–0.628)	0.001	0.478 (0.377–0.604)	<0.001	0.709 (0.453–1.112)	0.134
	Immunotherapy only	0.635 (0.191–2.108)	0.458	0.777 (0.100–6.039)	0.81	0.399 (0.219–0.730)	0.003	0.202 (0.048–0.856)	0.03
	Surgery + Radiation	0.126 (0.044–0.367)	<0.001	---	---	0.111 (0.045–0.277)	<0.001	0.517 (0.206–1.299)	0.161
	Surgery + Chemotherapy	0.126 (0.068–0.236)	<0.001	0.134 (0.051–0.353)	<0.001	0.168 (0.104–0.274)	<0.001	0.119 (0.034–0.421)	0.001
	Surgery + Immunotherapy	---	---	---	---	0.153 (0.037–0.626)	0.009	---	---
	Radiation + Chemotherapy	0.400 (0.216–0.742)	0.004	0.207 (0.079–0.542)	0.001	0.339 (0.221–0.520)	<0.001	0.541 (0.219–1.333)	0.182
	Radiation + Immunotherapy	0.307 (0.094–1.003)	0.051	---	---	0.372 (0.117–1.185)	0.094	---	---
	Chemotherapy + Immunotherapy	0.419 (0.145–1.212)	0.109	0.065 (0.008–0.518)	0.01	0.415 (0.152–1.132)	0.086	0.193 (0.025–1.468)	0.112
	Radiation + Chemotherapy + Immunotherapy	0.268 (0.036–1.981)	0.197	---	---	0.756 (0.104–5.481)	0.782	1.205 (0.155–9.370)	0.859
	Surgery + Chemotherapy + Immunotherapy	0.153 (0.062–0.379)	<0.001	0.247 (0.030–2.032)	0.193	---	---	---	---
	Surgery + Radiation + Chemotherapy	---	---	---	---	---	---	---	---
	Surgery + Radiation + Chemotherapy + Immunotherapy	0.182 (0.060–0.545)	0.002	---	---	0.133 (0.032–0.549)	0.005	---	---
Tumor Size	20–29 mm	1.50 (0.39-5.74)	0.556	0.43 (0.04-4.52)	0.481	1.49 (0.78-2.84)	0.225	0.91 (0.35-2.35)	0.840
	30–39 mm	1.33 (0.31-5.71)	0.706	0.26 (0.02-2.84)	0.269	1.81 (0.96-3.41)	0.068	1.93 (0.70-5.36)	0.207
	40–49 mm	1.87 (0.51-6.93)	0.348	1.69 (0.12-24.19)	0.698	1.14 (0.60-2.14)	0.695	2.99 (1.03-8.71)	0.044
	≥50 mm	0.65 (0.23-1.85)	0.417	0.71 (0.07-7.00)	0.768	1.95 (1.09-3.48)	0.024	1.55 (0.61-3.96)	0.358

--- indicates inability to generate a reliable hazard ratio due to small sample size, insufficient events, or model instability. Reference groups for the analysis were AFP < 400 ng/mL, female sex, stage I disease, no treatment, and tumor size < 20 mm for each respective category.

## Data Availability

This study utilized the National Cancer Database Liver PUF file for analysis, which is provided by the American College of Surgeons and can be accessed via application.

## Abbreviations

The following abbreviations are used in this manuscript:

HCC	Hepatocellular Carcinoma
NOS	Not Otherwise Specified
AFP	Alpha-Fetoprotein
NCDB	National Cancer Database
ICD	International Classification of Diseases
SEER	Surveillance, Epidemiology, and End Results Program

## Appendix A

**Table A1:** Predefined categories for AFP values recorded from 1998 to 2017.

Code	Description
000	0 ng/mL
001	1–19 ng/mL
002	20–29 ng/mL
003	30–39 ng/mL
004	40–49 ng/mL
005	50–59 ng/mL
006	60–69 ng/mL
007	70–79 ng/mL
008	80–89 ng/mL
009	90–99 ng/mL
010	100–199 ng/mL
020	200–299 ng/mL
030	300–399 ng/mL
040	400–499 ng/mL
050	500–599 ng/mL
060	600–699 ng/mL
070	700–799 ng/mL
080	800–899 ng/mL
090	900–999 ng/mL
100	1000–1999 ng/mL
120	2000–2999 ng/mL
130	3000–3999 ng/mL
140	4000–4999 ng/mL
150	5000–5999 ng/mL
160	6000–6999 ng/mL
170	7000–7999 ng/mL
180	8000–8999 ng/mL
190	9000–9999 ng/mL
200	Greater than or equal to 10,000 ng/mL
997	Test ordered, results not in chart
998	Test not done (not ordered and not performed)
999	Unknown or blank
